# Predicting Success: A Comprehensive Analysis of High School and Admission Test Scores on Future Academic Performance of Dental Students

**DOI:** 10.7759/cureus.56279

**Published:** 2024-03-16

**Authors:** Sana Iqbal, Amber Kiyani, Manahil Niazi, Faisal S Malik, Muhammad Humza Bin Saeed, Ulfat Bashir

**Affiliations:** 1 Department of Dental Education, Riphah International University, Islamabad, PAK; 2 Department of Dentistry, Riphah International University, Islamabad, PAK

**Keywords:** correlation, dental education, dental school performance, admission test score, high school score

## Abstract

Introduction

Dental school admissions in Pakistan traditionally rely on Higher Secondary School Certificate (HSSC), University of Health Sciences (UHS), and National Testing Service (NTS) scores, with limited research available on their predictive validity for dental school performance. This study aims to investigate the correlation between a student's first-year dental school performance and their HSSC, UHS, and NTS scores.

Methods

A total of 282 records, spanning the years 2016 to 2020, were obtained from a single private dental institution. The data included HSSC, UHS, and/or NTS scores, with the first professional examination results as the dependent variable. Statistical analysis was conducted using the Statistical Package for the Social Sciences (IBM SPSS Statistics for Windows, IBM Corp., Version 25.0, Armonk, NY), encompassing descriptive statistics, Pearson's correlation coefficients, and multiple regression analysis.

Results

Pearson's coefficients revealed weak to moderate positive correlations between the first professional examination and HSSC (r=0.209, p<.01), UHS (r=0.344, p<.01), and NTS (r=0.350, p<.01), all statistically significant at p < 0.01. Multiple regression analysis indicated that UHS scores contributed the highest explanatory power (R² = 0.146) in predicting first professional examination results.

Conclusion

A positive correlation between HSSC, UHS, and NTS scores with dental students' performance in the first professional examination is observed. However, the correlations are moderate, highlighting the importance of incorporating assessments that consider cognitive, behavioral, and skill-related aspects in admissions processes. Given the evolving landscape of dental education, these findings underscore the need for a holistic approach to identify candidates better equipped to serve the healthcare sector.

## Introduction

Dental schools have relied primarily on high school results and admission test scores for admission. An eligibility criterion is defined based on these two entities and used for screening candidates. Students who have the highest merit are eventually selected. This practice is common because it is speculated that the trend of hard work and intelligence will continue through medical or dental schools. It is also supported by some prior studies that demonstrate that prior educational performance and admission test results are reliable predictors of a student’s performance in medical school [1,2]. However, recent literature is beginning to challenge this notion.

A study from Jordan demonstrated a weak association between high school grades and admission tests with academic performance in medical school. However, it did predict the likelihood of completing medical school [3]. Another study from the United Kingdom (UK) showed that secondary school grades were inversely related to academic performance in the early years of medical school [4]. Similarly, an older study from the United States on dental school admissions asserted a strong relationship between dental school performance with dental admission tests and prior educational results, a recent study concluded that grade point average of previous education, results of pre-medical sciences, and dental admission test scores have a weak correlation with first-year dental school performance [5,6]. These results suggest that the applicability of current standards may vary from population to population and may have undergone change over the years.

Literature has demonstrated that admission test performance depends on socioeconomic status, gender, student confidence, and ability to perform under pressure. Therefore, it may not be a true reflection of the student’s prior or future performance. Previous literature has shown that male students are better test takers. These studies also show that women’s performance deteriorates under pressure and that they are more likely to avoid applying to schools that mandate entry examinations. In addition, socioeconomic status significantly impacts performance in aptitude tests [7-9]. These factors add a lot of bias to admission test scores. Taking this into consideration, Western institutions now employ multiple assessment strategies for entrance purposes. These include structured application forms, personal statements, interviews, mini-interviews (MMI), personality tests, and situational judgment tests (SJTs) to assess candidates [10]. Despite the stringent application of each of these entities, the selection of appropriate candidates remains challenging.

The Pakistani medical and dental school admission process remains traditional, relying exclusively on higher secondary school certificates (HSSC), which are the high school results and admission tests that can either be taken through the University of Health Sciences (UHS) or the National Testing Service (NTS). The combination of these scores is considered while formulating the merit criterion. Students qualifying for the merit are considered for admission. The effectiveness of this selection criterion also presents with contradictory results as prior studies from Pakistan have shown that admission tests and HSSC scores can be a reliable predictor of a student’s performance in the early years of medical school [11,12]. However, evidence of weaker correlations between the two is also available, along with recommendations to include non-cognitive assessments like interviews as admission prerequisites [13,14]. However, all these conclusions are based on investigations involving medical schools.

While the current research evidence on the relative strengths of admissions tests and other selection tools for medical school entry in Pakistan is marked by variations in both quality and quantity, the correlation of the same criteria for dental schools remains unknown. Given the unique challenges faced by Pakistan as a third-world country, ensuring efficient utilization through optimization of the selection processes. This is why it is important to ensure that the system, currently in use, is effective. Therefore, the objective of the investigation is to examine the correlation between a student's performance during the first year of dental school in Pakistan and the admission criteria currently used, specifically focusing on HSSC, UHS, and NTS results. Our results will provide valuable information regarding the efficacy of our admission process and can benefit government agencies, like the Pakistan Medical and Dental Council (PMDC) for improvements in this process.

## Materials and methods

This study was approved by the Ethical Review Board of Riphah International University (IIDC/IRC/2022/006/001). A retrospective cohort study design was used to analyze the correlation between the academic performance of students enrolled in a single private dental institute and their prior educational scores, covering a period from 2016 to 2020. The total duration of the study was six months (October 2021 to March 2022). The data of all students enrolled between the years 2016 and 2020 was accessed from the students’ private records and admittance histories, whereas the information about academic performance in the Bachelor of Dental Surgery (BDS) program was attained from the exams branch of the university after obtaining official permission. During this period, the curriculum underwent no substantial enhancements. All first-year students were enrolled in a standardized curriculum throughout this duration. Given that the initial year constitutes the exclusive pre-clinical phase, with assessments predominantly focusing on cognitive aspects, we utilized its outcomes for comparison with the cognitive components of the HSSC, NTS, and UHS examinations to ensure consistency in our analysis.

The inclusion criteria included students who had (1) appeared in the first professional examination conducted in the years 2017 to 2021, (2) had their HSSC scores available, and (3) had appeared in either of the NTS and/or UHS examinations. Any student with incomplete or alternate records for any category or had failed to conclude the course due to any reason or was considered ineligible to sit for the professional exam, repeated the year and took the exam with the next batch, or had to take re-sit examinations was excluded. The sample consisted of data from 375 students from the cohort of students admitted to the BDS program. The data was collected by universal sampling technique by two investigators, which a third investigator then cross-checked. All the identifiable information was discarded before the analysis of the data. The dependent variable was defined as the student's academic performance, i.e., the percentage in the first professional examination in the years 2017 to 2021. Independent variables included HSSC, NTS, and/or UHS scores. All values were entered as percentages to maintain uniformity for analysis. Data was analyzed using the Statistical Package for the Social Sciences (IBM SPSS Statistics for Windows, IBM Corp., Version 25.0, Armonk, NY). The data was presented as descriptive statistics, and Pearson correlation was determined to assess the predictive validity of the HSSC, UHS, and NTS exams for the student's academic performance. A P-value equal to or less than 0.05 was considered statistically significant. A linear regression model was designed to see the effect of HSSC, NTS, and UHS scores on the student's academic performance, i.e., the percentage obtained in the first professional exam.

## Results

Following the implementation of the exclusion criterion, 282 student records were retained. The mean HSSC, UHS, NTS, and first professional examination results are summarized in Table 1.

**Table 1:** Descriptive Statistics of Scores of HSSC, UHS, NTS, and First Professional Scores HSSC = Higher Secondary School Certificate, UHS = University of Health Sciences, NTS = National Testing Service

Marks (100%)	n	Mean (SD)	Skewness (SE)	Kurtosis (SE)
HSSC	282	82.6(4.15)	-0.31(0.15)	-0.15(0.29)
UHS	95	69(6.67)	-0.5(0.25)	0.64(0.49)
NTS	203	70.1(6.98)	-0.69(0.17)	2.03(0.34)
First Professional	282	70.5(6.82)	0.09(0.15)	-0.43(0.29)

Table 1 provides descriptive statistics for the scores of the HSSC, the UHS examination, the NTS examination, and the results of the first professional examination. The sample size (n), mean, standard deviation (SD), skewness, and kurtosis are shown in Table 1.

Table 2 presents the Pearson coefficients of correlation between the percent scores of HSSC, UHS, and NTS, and the first professional examination. The correlation between HSSC and UHS is 0.157, indicating a weak positive relationship. The correlation between HSSC and NTS is 0.256 (significant at the 0.01 level), suggesting a moderate positive correlation. Furthermore, the correlation between UHS and NTS is 0.451, indicating a moderate to strong positive relationship. Finally, the correlation between the first professional and HSSC, UHS, and NTS were 0.209, 0.344, and 0.350, respectively. These correlations, all significant at the 0.01 level, highlight a positive association between the scores of these educational components.

**Table 2:** Pearson Coefficient of Correlation Between Scores of HSSC, UHS, NTS, and First Professional Scores **p<0.01 HSSC = Higher Secondary School Certificate, UHS = University of Health Sciences, NTS = National Testing Service

Study Variables	1	2	3	4
HSSC	-			
UHS	.157	-		
NTS	.256**	.451	-	
First Professional	.209**	.344**	.350**	-

Table 3 outlines the outcomes of three regression models (Model 1, Model 2, and Model 3) investigating predictors of the final score for the first professional examination. In Model 1, HSSC exhibits a significant positive relationship with the final score, as evidenced by a β value of 0.21, a t-value of 3.57, and a p-value of less than 0.001. However, in Model 2, the significance diminishes for HSSC (β = 0.17, t = 1.73, p = 0.087), while the inclusion of the UHS variable reveals a significant positive relationship (β = 0.32, t = 3.25, p = 0.002). Model 3 retains significance for UHS (p = 0.000) and introduces the NTS variable with a significant positive coefficient (β = 0.32, t = 4.73, p < 0.001). Nevertheless, HSSC becomes nonsignificant (β = 0.11, t = 1.6, p = 0.111). Model 2 demonstrates the highest R2 (0.146), indicating increased explanatory power compared to Model 1 (0.044) and Model 3 (0.134) in predicting the final score for the first professional examination.

**Table 3:** Summary of Regression Analysis: Predictor of First Professional Score HSSC = Higher Secondary School Certificate, UHS = University of Health Sciences, NTS = National Testing Service

	Model 1			Model 2			Model 3	
	β (t-value)	P		β (t-value)	p		β (SE)	p
HSSC	0.21(3.57)	< .001>		0.17(1.73)	.087		0.11(1.6)	.111
UHS				0.32(3.25)	.002			
NTS							0.32(4.73)	.000
R^2^	0.044			0.146			0.134	

## Discussion

The PMDC mandates that the merit list generated for admissions by medical and dental schools reflects students’ performances in high school and entrance tests organized by the UHS and NTS. Owing to the lack of literature documenting the efficacy of the selection criteria in the literature, this study was designed to determine an association between academic performance during their first year of dental school and their high school score and entrance examination results.

We found a weak to moderate positive correlation between HSSC and the entrance examination. This is contrary to some of the evidence found in the literature that concludes that students with inferior academic performance sometimes are better test takers. These studies also show that male candidates perform better in admission tests despite inferior academic performance due to their confidence and their better handling of stress [8,9]. However, in Pakistan, the situation appears different. Women favor medical and dental professions in Pakistan and account for the majority of admissions in these schools. It is estimated that about 80% of enrolments in Pakistani medical/dental schools are female students [15]. This discredits the notion that male candidates are more likely to fulfill admission requirements.

We also concluded a strong positive relationship between NTS and UHS entrance examinations. Given that these are two separate exams conducted by two distinct agencies, based on these results we can conclude that the two test scores can be used interchangeably for admission in medical and dental schools across Pakistan. These results provide the first scientific evidence for the PMDC to accept multiple versions of the entrance examinations for admissions. We know from a study in the UK that multiple tests can be used for medical and dental admissions. A study from the UK concluded that results from the UK Clinical Aptitude Test (UCAT), the BioMedical Admissions Test (BMAT), and the Medical College Admission Test (MCAT) were somewhat comparable [16].

In addition, our results demonstrated a significant positive relationship and a weak correlation between the performance in the first professional examination and HSCC, although a subject-wise analysis was not carried out. Prior studies support our results. A study from Jordan also demonstrated a weak correlation between high school grade point average and performance in dental school [17]. Another study noted a minimal effect of high school results on academic performance through medical school. Still, it determined that students with exceptional high school performance were more likely to graduate on time [3]. Our conclusions and those from other similar studies seem logical because a hardworking student in high school will be expected to work hard during dental school as well.

However, sufficient evidence to the contrary is also present. A Saudi study determined that high school grades are not a good predictor of academic performance in medical school [18]. Similarly, a study from the UK concluded that undergraduate performance was inversely related to secondary school performance [19]. These studies show that solely dependent on academic performance and admission test scores can be unsatisfactory at times and create the need for a more thorough assessment.

With respect to the admission tests, we found a moderate positive correlation with academic performance. Prior studies have also demonstrated an association between academic performance and admission test scores. These studies concluded that admission test scores can be reliable predictors of academic success and the likelihood of graduation [3,6,20,21]. However, it is important to take into consideration that test performance can be affected by a magnitude of factors. These can include gender, socioeconomic status, and the ability to perform under pressure [7-9]. These admission test biases can exclude some potentially great dentists from entering dental schools.

We believe that our study can influence policy reform at both the institutional and national levels. This can include refining admission criteria, making it more equitable and accessible to a diverse applicant pool.

The strengths of this study include the use of a significant data set of high school scores, UHS, and NTS entrance exams over a five-year period. This ensures that the findings are evidence-based, enhancing the study's reliability and validity. Additionally, it reinforces the validity of using entrance exams as predictors of academic success. By highlighting the moderate positive correlation between admission test scores and academic performance, this study supports the ongoing use of such tests in the admissions process. It also acknowledges the need for broader assessment tools that include non-academic factors to identify candidates who are not only academically prepared but also possess the personal and professional qualities necessary for success in the dental field. It also addresses a significant gap in the literature regarding the efficacy of selection criteria in dental education, particularly in the context of Pakistan.

However, several limitations need to be addressed. The study focuses solely on cognitive predictors of success, neglecting non-cognitive factors such as motivation, resilience, and interpersonal skills. Future research should aim to incorporate these predictors. Another limitation of this study is sampling from a single dental institution. While this made our data collection process ethically and physically easier, it did include confounders such as the teaching ability of our faculty and the appropriateness of our learning environment. In addition, other confounding variables like study habits, socioeconomic background, and internal motivation were not considered during the analysis. This study also primarily focuses on first-year dental school performance, without considering long-term academic success or career outcomes.

Future research could benefit from including a larger and more diverse sample of dental schools across Pakistan. This would enhance the generalizability of the findings and provide a more comprehensive understanding of the relationship between entrance examinations, high school scores, and dental school performance nationwide. Additionally, they could include comparisons with dental schools in other countries could offer valuable insights into the efficacy of different admission systems. Longitudinal studies assessing student performance from the point of their entrance into dental school through graduation could provide a more comprehensive understanding of the predictive validity of high school and entrance examination scores in the longer term. Such studies could also help identify at what stages interventions might be most effective. Furthermore, researching the effectiveness of academic support and intervention programs for students identified as at risk based on their entrance test scores could help improve their retention and success rates. This could also inform the development of more targeted support services.

## Conclusions

In conclusion, this study demonstrates a positive correlation between HSSC, UHS, and NTS scores with dental students' performance in the first professional examination. However, the correlations are moderate, at best, highlighting the need for improvement. These results can assist in re-evaluating the traditional admission criteria to include a broader range of factors to assess a candidate’s potential and capabilities and provide evidence for refinement of the admission process defined by the PMDC thus fostering a more effective and equitable system by contributing to the enhancement of dental education in the country.

## References

[1] Kreiter CD, Axelson RD (2013). A perspective on medical school admission research and practice over the last 25 years. Teach Learn Med.

[2] Julian ER (2005). Validity of the Medical College Admission Test for predicting medical school performance. Acad Med.

[3] Tamimi A, Hassuneh M, Tamimi I, Juweid M, Shibli D, AlMasri B, Tamimi F (2023). Admission criteria and academic performance in medical school. BMC Med Educ.

[4] Mwandigha L. M., Tiffin P. A., Paton L. W., Kasim A. S., & Böhnke J. R. (2018). What is the effect of secondary (high) schooling on subsequent medical school performance? A national, UK-based, cohort study. BMJ Open.

[5] Rowland KC, Rieken S (2018). Rethinking dental school admission criteria: correlation between pre-admission variables and first-year performance for six classes at one dental school. J Dent Educ.

[6] Sandow PL, Jones AC, Peek CW, Courts FJ, Watson RE (2002). Correlation of admission criteria with dental school performance and attrition. J Dent Educ.

[7] Lambe P, Waters C, Bristow D (2012). The UK Clinical Aptitude Test: is it a fair test for selecting medical students?. Med Teach.

[8] Finger C, Solga H (2023). Test participation or test performance: why do men benefit from test-based admission to higher education?. Sociol Educ.

[9] Montolio D, Taberner PA (2021). Gender differences under test pressure and their impact on academic performance: a quasi-experimental design. J Econ Behav Organ.

[10] Cox WC, Wolcott M, Hahn F, McLaughlin JE (2023). The relationship between a multiple mini-interview and situational judgment test for admissions. Am J Pharm Educ.

[11] Iftikhar S, Cheema KM, Aziz I (2023). MBBS admission criteria as predictor of academic performance in a medical college of Pakistan. Pak J Med Sci.

[12] Khan IZ, Mahboob U, Yasmeen R (2022). Correlation between admission criteria and academic performance in medical and dental colleges of Khyber Pakhtunkhwa. Pak J Med Sci.

[13] Luqman M (2013). Relationship of academic success of medical students with motivation and pre-admission grades. J Coll Physicians Surg Pak.

[14] Qazi MA, Anwar J, Moffat M (2020). Predictive ability and stakeholders’ perceptions of the selection tools for MBBS in women medical College: a mixed methods study. J Ayub Med Coll Abbottabad.

[15] Raza A, Jauhar J, Abdul Rahim NF, Memon U, Matloob S (2023). Unveiling the obstacles encountered by women doctors in the Pakistani healthcare system: a qualitative investigation. PLoS One.

[16] Davies DJ, Sam AH, Murphy KG, Khan SA, Choe R, Cleland J (2022). BMAT's predictive validity for medical school performance: a retrospective cohort study. Med Educ.

[17] Al-Asmar AA, Oweis Y, Ismail NH, Sabrah AH, Abd-Raheam IM (2021). The predictive value of high school grade point average to academic achievement and career satisfaction of dental graduates. BMC Oral Health.

[18] Guraya SF, Zolaly MA (2012). High school grades are not reliable predictors of academic performance in undergraduate medical school: a study from a Saudi Medical School. Biomed Pharmacol J.

[19] Mwandigha LM, Tiffin PA, Paton LW, Kasim AS, Böhnke JR (2018). What is the effect of secondary (high) schooling on subsequent medical school performance? A national, UK-based, cohort study. BMJ Open.

[20] Althewini A, Al Baz N (2022). Prediction of admission tests for medical students’ academic performance. Adv Med Educ Pract.

[21] Migliaretti G, Bozzaro S, Siliquini R, Stura I, Costa G, Cavallo F (2017). Is the admission test for a course in medicine a good predictor of academic performance? A case-control experience at the school of medicine of Turin. BMJ Open.

